# Preferences Among Expert Physicians in Areas of Uncertainty in Venous Thromboembolism Management: Results from a Multiple-Choice Questionnaire

**DOI:** 10.3390/jcm14238531

**Published:** 2025-12-01

**Authors:** Alessandro Di Minno, Gaia Spadarella, Ilenia Lorenza Calcaterra, Antonella Tufano, Alessandro Monaco, Franco Maria Pio Mondello Malvestiti, Elena Tremoli, Domenico Prisco

**Affiliations:** 1 Department of Pharmacy, Federico II University Naples, Via D. Montesano 49, 80138 Napoli, Italy; 2 CEINGE-Biotecnologie Avanzate, Via Gaetano Salvatore 486, 80131 Naples, Italy; 3 Dipartimento di Scienze Biomediche Avanzate, Federico II University Naples, 80138 Napoli, Italy; 4 Dipartimento di Medicina Clinica e Chirurgia, Università degli Studi di Napoli Federico II, 80138 Naples, Italyatufano@unina.it (A.T.); 5 Oliba Healthcare and Pharma, 00192 Rome, Italy; 6 Viatris, 00144 Rome, Italy; 7 Maria Cecilia Hospital, 48033 Cotignola, Italy; etremoli@gvmnet.it; 8 Department of Experimental and Clinical Medicine, University of Florence, 50121 Florence, Italy; domenico.prisco@unifi.it

**Keywords:** hospitalized medical persons, antiplatelet agents, subsegmental pulmonary embolism, post-pulmonary embolism syndrome, outpatient treatment

## Abstract

**Background/Objectives**: Prevention and treatment of venous thromboembolism (VTE), including deep vein thrombosis (DVT) and pulmonary embolism (PE), is a major clinical issue in hospitalized patients. Some aspects of VTE management lack clarity due to differing physicians’ opinions and behaviors. **Methods**: A multidisciplinary steering committee identified two main areas of uncertainty: VTE prophylaxis and PE management in special settings. A multiple-choice questionnaire including 10 statements was circulated to 183 doctors trained in VTE management. The expected benefit-to-harm ratio was represented on a nine-point Likert scale, with consensus (≥75% agreement) on scores of 1–3 indicating inappropriate and 7–9 indicating appropriate care measures. **Results**: In online voting, a consensus was reached for 9/10 statements. Respondents considered the following to be appropriate: risk assessment of VTE (93.44%) and bleeding (91.6%) in hospitalized medical patients; low-molecular weight heparin (LMWH) prophylaxis for inpatients with pneumonia and malignancy (82.78%); therapeutic doses of LMWH/fondaparinux in patients with intermediate/high risk of PE with (80.9%) or without (77.97%) instability criteria; and echocardiography to manage patients with a post-PE syndrome (93.99%). Respondents considered the following to be inappropriate: use of 4000 IU LMWH in chronic renal failure (80.46%); use of 2000 IU LMWH in persons on dual antiplatelet therapy (77.01%); and use of low-dose apixaban (2.5 mg) in pregnancy (88.57%) or in subsegmental PE with hypoxemia (82.46%). No consensus was reached on the identification of PE cases eligible for outpatient treatment. **Conclusions**: Our findings show persistent gaps between guideline recommendations and clinical implementation despite improved awareness among physicians. Uncertainty persists regarding criteria for outpatient PE eligibility and/or for validation of bleeding-risk models.

## 1. Introduction

Compared with residents in the community, hospitalized older individuals have a >130-fold higher incidence of venous thromboembolism (VTE; 71 vs. 9605 cases/100,000 person-years) [1]. Because of concomitant morbidities (e.g., stroke, coronary artery disease), antiplatelet drugs are chronically used in most hospitalized older individuals. Despite comprehensive evidence-based guidelines, thromboprophylaxis may promote bleeding in chronic antiplatelet users [1]. Ad hoc risk assessment models (RAMs) have been developed to predict a person’s risk of bleeding and of VTE recurrence. However, commonly used RAMs have a weak predictivity in hospitalized patients [2]. In users of low-molecular weight heparin (LMWH) plus antiplatelet agents, uncertainties in the risk of bleeding may arise from publication bias [3]. Various definitions of major and fatal bleeding in different clinical studies also lead to conflicting classifications and controversial estimates of patient risk [4,5]. Other uncertain issues relate to the management of VTE patients. Improved knowledge of management of cancer-related VTE [6], subsegmental pulmonary embolism (PE) [7], post-PE syndrome [8], people with intermediate/high risk of PE who do/do not meet the hemodynamic instability criteria [9], as well as newer information concerning the identification of PE cases eligible for outpatient treatment [10], raise additional issues about optimal VTE handling. To identify persistent discrepancies between guideline recommendations and clinical implementation, we surveyed 183 expert physicians about 10 controversial issues in VTE prophylaxis and management.

## 2. Methods

In 2023, the Giovanni Lorenzini Medical Foundation planned to assess, via ad hoc surveys, current attitudes, beliefs, and unaddressed questions related to prevention and cure of VTE. As VTE handling requires a multidisciplinary approach involving medical doctors, nurses, pharmacists, and other healthcare professionals, separate surveys were planned for the different healthcare professionals. The general database of the Lorenzini Foundation, which holds email addresses of approximately 50,000 associates, 20,000 of whom are physicians, served as a resource for the first survey targeting medical doctors. A multidisciplinary steering committee (consisting of a pharmacologist, an internist, and a hematologist experienced in different aspects of VTE management) was established.

Controversial issues in the area were identified with a literature search using the Medline, Scopus, Web of Science, and Cochrane databases according to validated methods. The following keywords (in the title/abstract) were employed, with no date or language limits: “thromboprophylaxis” OR “prophylaxis” OR “venous thromboembolism” OR “VTE” OR “thromboembolism” OR “heparin” OR “low-molecular-weight heparin” OR “LMWH” OR “PE” OR “aspirin” OR “warfarin” OR direct anticoagulant drugs, AND “antiplatelet treatment” OR “antithrombotic drugs” OR “risk assessment method” OR “RAMs” OR “hospitalized” OR “awareness” OR kidney failure; OR “pre-eclampsia” OR “postpartum” OR “pregnant” OR “preeclampsia” OR “eclampsia”.

To capture the perspectives of a large group of doctors active in VTE prevention and treatment, a multiple-choice questionnaire was planned to explore current practice and identify the challenges faced by doctors when managing subjects with, or at risk of, VTE. Prior to drafting the ad hoc questionnaire (June 2023), two virtual presentations were provided by the steering committee (May 2023) to review and debate challenges in VTE management that are encountered in clinical practice. Questions and issues raised by attendees at these sessions were used to inform a 10-statement multiple-choice questionnaire that was drafted by the steering committee. The content of the statements was independently compared by members of the steering committee with established guidelines on VTE management (e.g., Royal College of Obstetricians and Gynaecologists [RCOG] guidance, 2019; European Society of Cardiology [ESC] 2019; American Society for Hematology [ASH] 2020).

To validate the survey instrument and ensure the suitability of the questionnaire (i.e., clarity, relevance, and difficulty), a pilot survey was conducted among a small group of young doctors working in the Internal Medicine and the Clinical Medicine wards at two academic hospitals (Federico II, Naples [n = 15]; Careggi, Florence [n = 15]). No changes were made to the questionnaire following positive feedback from the pilot group.

Using information from the Giovanni Lorenzini Medical Foundation database, the survey and instructions for completion were emailed to physicians from the following specialties: Internal Medicine, Cardiology, Geriatrics, Neurology, Orthopedics, and Vascular/General Surgery (N = 257). Many of the recipients had attended the two online presentations (as had the doctors who completed the pilot survey). To facilitate consultation on the VTE issues being addressed, survey recipients (pilot and final) also received a list of references supporting the rationale underlying statements and providing information on key populations, diagnostic tools, and pharmacological and non-pharmacological treatment options. (Table S1).

A modified Delphi method was employed by the steering committee to assess the results of the survey [11]. For the consensus process, participants expressed their opinion on the level of expected benefit-to-harm on a nine-point Likert scale, with management steps considered inappropriate having scores of 1–3 and those considered appropriate having scores of 7–9; a score of 4–6 reflected a comparable harm–benefit profile. A value of 1 was considered to reflect where expected harm(s) offset any expected benefit(s), and vice verse for a value of 9. An option not to respond to a question was also possible. In March 2024, the steering committee analyzed the responses and synthesized the data. Consensus was achieved when ≥75% of responses were in the 7–9 (“appropriate”) or 1–3 (“inappropriate”) ranges (Table S1).

## 3. Results

Among the 257 doctors within the selected medical specialties who received the link and related instructions, 183 completed the survey, of which 182 responses were deemed valid. Of these, 158 responses were from doctors from Italy, 7 from Portugal, 3 from Spain, 2 from France, 2 from Greece, and 1 from each of the following: Germany, Poland, Bulgaria, the UK, Lithuania, Algeria, the United Arab Emirates, Brazil, Argentina, Chile, and Australia. Details of survey participants, including medical specialty, years of experience, and country of practice are presented in Table 1. No notable variations or major inconsistencies in responses were observed based on respondent specialty or geography.

In online voting, a consensus (i.e., ≥75% of ratings within the Likert scale 1–3 or 7–9) was reached in 9/10 statements (Table 2). In particular, the following options were considered appropriate by the participants: risk assessment of VTE (93.44%) and of bleeding (91.6%) in hospitalized medical patients; prophylactic doses of LMWH for a patient with malignancy hospitalized for pneumonia (82.78%); therapeutic doses of LMWH/fondaparinux for the acute treatment of patients with intermediate/high-risk (80.9%) or intermediate/low-risk (77.97%) PE; and echocardiogram evaluations to manage patients with a post-PE syndrome (93.99%). In contrast, participants considered the following options to be inappropriate: 4000 U IU LMWH for thromboprophylaxis in chronic renal failure (80.46%); low-dose thromboprophylaxis (2000 IU LMWH) in dual antiplatelet therapy (77.01%); low-dose apixaban (2.5 mg) in pregnancy (88.57%); and prophylactic dose of fondaparinux/LMWH in patients with subsegmental PE and hypoxemia (82.46%). Only 70.93% of participants agreed on the identification of PE cases eligible for outpatient treatment; thus, no consensus was reached on this statement (i.e., <75% agreement).

## 4. Discussion

Using a modified Delphi approach, we surveyed 183 expert physicians regarding 10 areas of uncertainty in VTE prophylaxis and management. Consensus was reached on 9 of the 10 statements, indicating that participants had a relatively consistent approach to addressing most of the issues presented.

### 4.1. Assessing Risk of VTE and Bleeding in Hospitalized Medical Patients

There was strong consensus among participants on the opportunity to assess the risk of VTE and of bleeding in all patients entering an Internal Medicine ward. In 2008, the cross-sectional ENDORSE survey reported that, out of the 15,487 medical patients at risk for VTE (according to the American College of Chest Physicians [ACCP] criteria), only 6119 (40%) received ACCP-recommended VTE prophylaxis [12]. In 2022, among 137,288 acutely ill medical patients, suitable thromboprophylaxis was administered to ≈54% of subjects with an indication. Active bleeding, renal dysfunction, and thrombocytopenia were major factors in deciding against thromboprophylaxis [13]. Several RAMs (e.g., CAPRINI risk model, Korana risk score, PADUA risk score, RCOG risk score, IMPROVE risk score, IMPROVE D-dimer, GENEVA risk score) have been developed to assess adherence to guideline recommendations [14]. These RAMs can also help to tailor thromboprophylaxis by identifying medical patients who would most benefit from it in hospital and post-discharge [2], as well as those who should avoid unnecessary anticoagulation due to an increased risk of bleeding [15]. The Padua Prediction score and the ACCP criteria have been often adopted to predict VTE risk [13]. In contrast, none of the studies examined in the systematic review (n = 27, 2010–2020) used the IMPROVE Risk Score (Table S2) to identify patients at high risk of bleeding [16]. The comparative accuracy of some RAMs (i.e., the Caprini risk model, the Padua risk score, the IMPROVE models, the Geneva risk score) and of other scoring systems to predict the risk of developing VTE in hospitalized patients has been systemically reviewed [2]. Of the 51 studies included in the review, 21 were devoted to hospitalized patients who required medical care. The authors concluded that due to uncertainty regarding the accuracy of factors affecting risk assessment and tailored pharmacological prophylaxis, all RAMs have weak predictive accuracy for VTE, and there is weak evidence and too much heterogeneity to recommend the practical use of any RAM [2].

### 4.2. VTE Prophylaxis in an Elderly Patient with Stage IV Chronic Renal Failure

Participants considered the administration of 4000 IU LMWH inappropriate for thromboprophylaxis in chronic renal failure, in line with guidelines.

The other suggested management options were chosen by only 50–65% of participants. Approximately 40% of medical patients have moderate–severe chronic kidney disease (CKD) [17]. Prophylaxis of VTE is challenging because of CKD-related and drug-related risk of bleeding. In a systemic review of 674 publications on this topic, no accumulation was observed in studies using dalteparin or tinzaparin. In contrast, prophylactic doses of enoxaparin, bemiparin, or certoparin did accumulate in patients with a creatinine clearance (CrCl) below 30 mL/min. No information was available for nadroparin. Accordingly, the authors recommended dose reduction when using prophylactic LMWHs (apart from tinzaparin and dalteparin) in renal insufficiency [18]. Additional evidence supports the use of dalteparin in severe CKD [19], but there are limited data on the use of other LMWHs in severe CKD. Consequently, the prophylactic dose of LMWHs other than dalteparin should be reduced by 50% (Table S3). In addition, anti-Xa activity should be controlled in those with CrCl < 30 mL/min who are treated with LMWH. As unfractionated heparin (UFH) is metabolized in the liver, it is the drug of choice in severe CKD, using prophylactic doses and periodically checking activated partial thromboplastin time (aPTT). Mechanical prophylaxis may be considered as an alternative to heparins. Both atrial fibrillation (AF) and VTE are highly prevalent in the elderly and in patients with CKD. Direct oral anticoagulants (DOACs) are recommended for the long-term treatment of AF and of VTE in non-hospitalized subjects because they may be used in fixed doses without routine monitoring, have few food and drug interactions, and are associated with a low risk of life-threatening bleeding [20]. Clinicians should use renal function estimates to inform the choice of DOAC [21]. However, DOACs are not indicated or licensed for use in VTE prophylaxis in medical inpatients [16].

### 4.3. Thromboprophylaxis in a Patient on Dual Antiplatelet Therapy (DAPT)

Survey participants considered it inappropriate to prescribe low-dose thromboprophylaxis (2000 IU LMWH) in patients receiving DAPT. This is probably based on the fact that enoxaparin trials for the VTE prophylaxis in Internal Medicine were performed with a daily dose of 4000 IU. Only 35% of doctors considered it appropriate to maintain DAPT when adding standard-dose enoxaparin; the majority of participants preferred to stop clopidogrel (64%) rather than aspirin (37%). It is likely that most participants considered this to represent a safer de-escalation of DAPT, although no formal consensus was reached on this point.

Thromboprophylaxis in patients receiving antiplatelet agents (alone or in combination) increases the risk of bleeding [22], and recommendations for perioperative minimization of the risk have been published (Table S4). In patients receiving DAPT who develop medical conditions and need LMWH/fondaparinux, the recommendations are to consider their bleeding risk on an individualized basis, with regular risk assessment over time [23].

### 4.4. Thromboprophylaxis in a Patient with Malignancy Hospitalized Because of Pneumonia

Participants considered prophylactic doses of LMWH appropriate for a patient with malignancy who was hospitalized for pneumonia. Most respondents were aware that DOACs are not indicated for prophylaxis out of orthopedic setting, but there was some confusion on the use of the Khorana score.

Cancer patients are at high risk for DVT and PE depending on both tumor site and disease stage. Surgery and systemic anticancer treatments further enhance patients’ thrombotic risk [6]. A prospective, cross-sectional study in hospitalized patients conducted at five academic hospitals to define prescription rates and factors affecting thromboprophylaxis during hospitalization concluded that despite relative contraindications, pharmacologic thromboprophylaxis is often administered to hospitalized cancer patients [24]. Nevertheless, although effective, thromboprophylaxis may be associated with enhanced risk of bleeding in cancer patients. However, the Khorana score, initially established for outpatients, (Table S5) has only been retrospectively validated in hospitalized cancer patients [25]. Hence, thromboprophylaxis may not be appropriately targeted and may not benefit cancer patients as a subgroup (Table S6). Randomized controlled trials are needed to assess the efficacy and safety of routine administration of parenteral thromboprophylaxis in hospitalized medically ill patients with cancer. Extended thromboprophylaxis with DOACs is not licensed post-discharge because their ability to lower VTE recurrence (compared with standard heparin prophylaxis) is offset by a high tendency to cause major bleeding [6].

Regarding thromboprophylaxis in a 21-week pregnant 35-year-old woman on low-dose aspirin for gestational hypertension hospitalized for ≥3 days for urgent clinical evaluations, participants only reached a consensus in excluding the use of apixaban in pregnancy, but consensus was not achieved for any of the other management options. This is likely due to an unclear definition of the risk in the presented case. The patient was considered at high risk by 55% of participants.

In addition to physiological adaptation to the hemostatic challenge of delivery, factors that increase the “physiological” background risk of VTE in pregnancy may be present prior to the pregnancy (e.g., maternal thrombophilia, prior VTE) or develop during the pregnancy (e.g., pre-eclampsia) or following delivery (e.g., infection) [26]. RCOG guidance, updated in 2019, recommends assessment of a woman’s risk of VTE at specific timepoints during pregnancy (i.e., pre-pregnancy, antenatally and at each subsequent contact or hospital admission, and postnatally at the time of delivery) [26]. Despite weak evidence [26], such guidance provides clinicians with an estimate of the impact of each risk factor during pregnancy and postnatally at the time of delivery; this helps set a framework to inform decision-making on antenatal and/or postnatal thromboprophylaxis with LMWH based on the identification and quantification of documented risk factors for VTE alone and in combination (Table S7). The rationale is that the higher the risk for VTE, the higher the benefit from prophylaxis. LMWH is recommended when VTE risk is elevated, with duration and dosage to be employed varying depending on the individual risk [27]. In addition to VTE prevention and management, LMWH administration also reduces the incidence of gestational hypertension, perinatal death, and preeclampsia in high-risk pregnant women and improves pregnancy outcomes [10,11] without increasing the risk of bleeding [28]. The same beneficial effects are observed using low-dose aspirin combined with LMWH [3]. However, data on intrapartum or postpartum bleeding related to the combined use of these two drugs was only reported in 3 of the 14 studies in the meta-analysis (i.e., in 233 of 1165 women) [3].

For the optimal treatment of a patient with intermediate–high risk PE (according to the ESC criteria) who does not meet the hemodynamic instability criteria, participants reached a consensus on the use of subcutaneous (sc) fondaparinux or LMWH at therapeutic dose, but only one in two respondents considered it appropriate to prescribe iv UFH or rivaroxaban. Surprisingly, ≈10% of participants considered thrombolysis to be appropriate. ESC guidelines recommend anticoagulation with parenteral (LMWH, fondaparinux) or oral DOACs in these patients, limiting the use of UFH to those at risk of hemodynamic decompensation or with severe renal impairment or severe obesity. According to the ESC, 30-day mortality in these patients is 10.9% [9]. The wide use of parenteral anticoagulation in this setting during hospitalization has been recently reported in a large Italian population [29]. It is unclear to what extent hemodynamically stable acute PE patients with a clinical parameter of PE severity (e.g., a simplified Pulmonary Embolism Severity Index [sPESI] > 0, signs of right ventricular dysfunction [RVD] and/or myocardial injury) benefit from reperfusion therapy. The use of systemic thrombolysis in this clinical setting was explored in the PEITHO study, which included 1005 patients with intermediate–high risk of acute PE with RVD on a CT pulmonary angiogram and a positive troponin test; participants were randomized to standard anticoagulation or anticoagulation plus a single-bolus injection of tenecteplase (30–50 mg, based on body weight) [30]. While it prevented death and/or hemodynamic decompensation (7-day incidence: 2.6% vs. 5.6%, odds ratio [OR] 0.44; 95% confidence interval [CI] 0.23–0.87) compared with a placebo, systemic tenecteplase administration was associated with a high risk of major extracranial bleedings (6.3% vs. 1.2%) and of hemorrhagic stroke (2.0% vs. 0.2%) However, a post hoc analysis of the trial showed that, in intermediate–high risk of acute PE with at least two clinical criteria of severity (i.e., systolic blood pressure [SBP] ≤ 100 mmHg, respiratory rate > 20 breaths/min, chronic heart failure, and/or cancer), tenecteplase addition resulted in an adverse event rate of ≈7% compared to ≈20% in the placebo PEITHO group [31]. While further risk stratification should help to select patients in whom thrombolysis could be attempted, low-dose thrombolysis is an ongoing approach to preserve a high rate of thrombus resolution with the lowest risk of bleeding and dying [32]. Additional approaches include the use of catheter-directed treatments (e.g., thrombus fragmentation, thrombus aspiration, rheolytic thrombectomy, or local ultrasound-accelerated thrombolysis).

### 4.5. Optimal Treatment in a Patient with Intermediate–Low Risk of PE According to ESC Criteria

Participants considered it appropriate to use sc fondaparinux or LMWH at a therapeutic dose (80.9%) in patients with intermediate–low risk of PE. Interestingly, 72% also considered it appropriate to use rivaroxaban 15 mg twice daily (bid), while only 31% considered edoxaban 60 mg once daily (od) appropriate.

The intermediate–low-risk PE category includes patients who have normal cardiac biomarker levels and/or in whom the right ventricle (RV) is normal on echocardiography or on pulmonary angiograms. Both patients with intermediate–low and intermediate–high risk of PE are usually hospitalized and receive the same treatments. However, clinical monitoring is less stringent in those at intermediate–low risk, and their discharge is usually quicker. In view of the implications of early discharge, additional stratification for PE risk in the absence of hemodynamic instability is recommended at presentation [9].

### 4.6. Acute Treatment of Patients with Subsegmental PE (SSPE) and Moderate Hypoxemia

Participants considered it inappropriate to use prophylactic doses of fondaparinux or LMWH in this setting, while 78% considered it appropriate to treat these patients with therapeutic doses of fondaparinux or LMWH; 64% would also consider rivaroxaban 15 mg bid.

Advances in computed tomography (CT) have increased interest in minute pulmonary emboli in subsegmental arteries (i.e., in SSPE). However, there is uncertainty about prognosis and VTE recurrence in this setting because of paucity of evidence from randomized clinical trials.

Selection of SSPE patients who can be untreated is critical (Table S8). A large multicenter prospective cohort study showed a recurrent VTE rate of 3.1% (8 out of 266 patients; 95%CI 1.6–6.1; none of the recurrences were fatal) in SSPE without DVT [7]. Since an isolated SSPE may become clinically relevant in patients with preexisting cardiopulmonary disease [33], common sense argues that SSPE patients presenting with hypoxemia need treatment. Likewise, since the expected recurrence rate is high even when the diagnosis is incidental, SSPE patients with a malignancy or a VTE history should be treated. Finally, since DVT predicts VTE recurrence and PE-related mortality, patients with SSPE and a DVT should be treated.

### 4.7. Managing a Patient Appropriately Treated for PE with Persistent Dyspnea

Participants considered it appropriate to perform echocardiograms in these patients. There was less agreement on employing a ventilation/perfusion (VQ) scan or cardiopulmonary stress test as an initial assessment.

After 3–6 months of adequate anticoagulation, some PE survivors have persistent symptoms and/or restrictions on daily activities unrelated to pre-existing comorbidities. Symptoms defining a post-PE syndrome (PPES) include persistent dyspnea, fatigue, exercise intolerance, and impaired quality of life in PE survivors. Additional symptoms may include chest pain, anxiety, and depression. Awareness is critical to identify and treat PPES among PE survivors [8]. Those who report symptoms of reduced functional status associated with measurable limitations in cardiopulmonary function have a post-PE impairment (PPEI) [8]. Measurable limitations include abnormalities in New York Heart Association score, 6 min walking distance echocardiography, VQ scan, and cardiopulmonary exercise test [34,35,36,37]. In a patient with persistent dyspnea and poor physical performance, the ESC algorithm recommends early trans-thoracic echocardiography to estimate the probability of chronic thromboembolic pulmonary hypertension (CTEPH). Clinical implications of PPEI and late outcomes of acute PE have been prospectively assessed in adult consecutive patients with confirmed acute symptomatic PE, followed up with a standardized assessment plan and pre-defined criteria and visits [38]. Co-primary outcomes of this study were diagnosis of CTEPH and PPEI, defined as worsening in severity or persistence of the highest severity compared with the previous visit. Of 1098 patients, 1017 (median age 64 years, 45% women) entered the primary CTEPH analysis. CTEPH (diagnosed in 16 [1.6%] patients, after a median of 129 days) had an estimated 2-year cumulative incidence of 2.3% (1.2–4.4%). The 2-year cumulative incidence of the PPEI (analyzed in 880 patients) was 16.0% (95% CI 12.8–20.8%) and helped identify patients diagnosed with CTEPH during the follow-up (HR for CTEPH vs. no CTEPH 393; 95% CI 73–2119). Compared with patients without, those with PPEI also had a higher risk of re-hospitalization and death and a worse quality of life. These data support the systematic evaluation of symptom burden and quality of life after acute PE to optimize algorithms and guideline recommendations in this setting, 39 in addition to facilitating the early identification of patients who need additional treatment beyond anticoagulation. Patient-reported outcome measures of dyspnea (using the Medical Research Council dyspnea scale) or functional limitations (using the Post-VTE Functional Status [PVFS] scale) should be used for this purpose. In the absence of symptoms and signs, risk factor assessment for CTEPH should be conducted, according to the ESC checklist [9]. Cardiopulmonary rehabilitation is a useful option for these patients [39].

### 4.8. Identifying PE Cases Eligible for Outpatient Treatment

No consensus (i.e., agreement < 75%) was reached on the identification of PE cases eligible for outpatient treatment. Few participants (30.8%) considered the Hestia criteria appropriate and most participants (70.93%) believed RVD required hospitalization regardless of the Hestia criteria.

Based on the 2019 ESC guidelines, a score of 9 in the sPESI index identifies low-risk PE patients eligible for outpatient treatment, and the absence of RVD is mandatory to define low-risk patients. Nonetheless, the Hestia criteria can help to identify the need for advanced reperfusion therapy or oxygen therapy, regardless of the sPESI score, in addition to providing an alternative tool to identify cases eligible for outpatient treatment [40]. In a prospective cohort study, patients who were negative for all the Hestia criteria (Table S9) were selected for outpatient treatment [10]. Among them, 35% were normotensive individuals with RVD and were classified as intermediate-risk according to ESC criteria. The 90-day overall mortality of home-treated patients according to the Hestia criteria was 1.0%. Adverse events occurred in 22 hospital-treated patients (4.5%) vs. none of the home-treated patients (*p* < 0.001). Thus, regardless of RVD, none of the hemodynamically stable patients treated at home had a PE-related adverse event in this study [10]. Negative predictive value for adverse outcome was 100% for the Hestia criteria and less for the ESC criteria. However, a meta-analysis of 3295 ‘low-risk’ patients with acute PE showed that RVD on admission is associated with early mortality (OR 4.19, 95% CI 1.39–12.58) [41]. Whether RVD or high troponin values improve identification of low-risk patients over clinical models alone was investigated. Studies assessing the relationship between RVD (or elevated troponin) and short-term mortality in patients with acute PE at low risk of death based on clinical models (PESI, sPESI, Hestia) were retrieved and data from 5010 low-risk patients were pooled. In an individual patient meta-analysis, 30-day mortality was 0.7% (95% CI 0.4–1.3), and RVD at echocardiography, CT, or B-type natriuretic peptide (BNP)/N-terminal pro BNP (NT-proBNP, BNP/NT-proBNP) values helped identify those with a high risk of short-term death (1.5 vs. 0.3%; OR 4.81, 95% CI 1.98–11.68). High troponin levels were also associated with short-term death (OR 2.78, 95% CI 1.06–7.26), and RVD and troponin values were associated with death within 3 months (RVD: 1.6 vs. 0.4%, OR 4.03, 95% CI 2.01–8.08; troponin OR 3.68, 95% CI 1.75–7.74). Thus, RVD by echocardiography or BNP/NT-proBNP helps identify candidates for outpatient management or short hospital stays [42].

## 5. Conclusions

Our findings align with previous surveys showing persistent gaps between guideline recommendations and clinical implementation, despite improved awareness among physicians. While survey results indicate that physicians’ preferences align with existing trial data and guidelines for most inpatient scenarios, uncertainty remains regarding thromboprophylaxis in CKD, pregnancy, and DAPT-treated patients. Uncertainty in specific scenarios highlights the need for targeted investigation and validation of bleeding-risk models. Accordingly, our findings highlight the need for prospective trials (e.g., to define outpatient PE eligibility and/or for validation of bleeding-risk models). Several trials are ongoing in the management of patients with PE. In those with intermediate–high risk PE (according to the ESC criteria) who do not meet the hemodynamic instability criteria, the PEITHO-3 trial (NCT04430569) is evaluating the efficacy and safety of a reduced-dose alteplase regimen plus standard heparin in patients with intermediate–high risk PE and at least one the following criteria of clinical severity: SBP < 100 mmHg; respiratory rate > 20 breaths/min, and chronic heart failure. In intermediate–high-risk PE patients with at least two of the following severity criteria, heart rate ≥ 100 beats/min, SBP ≤ 110 mmHg, respiratory rate > 20/min, or O_2_ saturation < 90% on room air, the HI-PEITHO trial (NCT04790370) is testing risk/benefit ratios of ultrasound-facilitated catheter-directed thrombolysis anticoagulation vs. anticoagulation alone. In SSPE patients randomized to a placebo or rivaroxaban, the safe-SSPE trial (NCT04263038) assesses the incidence of recurrent VTE, recovery, and functional performance [43].

The results of new trials will be critical to providing a high level of evidence and appropriate recommendations on evolving areas in VTE. Indeed, the present data also show that, like the optimal prevention of VTE recurrence (Table S10), routine administration of parenteral thromboprophylaxis in medical inpatients is perceived as a contemporary gray area. When anticoagulation is combined with antiplatelet treatment, doctors consider the risk of bleeding a major concern in VTE management [44]. Despite the growing general awareness of the risk of bleeding among those receiving antithrombotic drugs, there are limited data available on identifying individual at risk. The implications of the discrepancy between participants’ approaches to identifying PE cases eligible for outpatient treatment need to be further explored. Echocardiography-documented RVD is associated with death (but not with PE-related death) in stable patients and indicates the potential utility of echocardiography and laboratory markers in clinical assessment [45]. This is also consistent with the RV/LV diameter ratio and tricuspid annular plane systolic excursion (TAPSE) being used to measure such variables. Educational initiatives should emphasize universal use of structured VTE and bleeding-risk assessment tools (e.g., PADUA, IMPROVE) and encourage documentation of clinical rationale when management deviates from guidelines. Emerging evidence will also facilitate improved decision-making.

Finally, when evidence is limited, clinicians rely on additional patient-specific factors to guide care. In those settings, clinicians make individualized decisions based on additional information to suitably risk-stratify the individual patient and establish optimal treatment strategies [46]. In a program for persons undergoing percutaneous coronary interventions, which was implemented in nine large US centers, informing physicians of a patient’s bleeding risk led to a 44% reduction in the occurrence of bleeding [47]. Information may also stem from genomics, when individual profiles (e.g., polymorphisms) become more widely available. Individual patient factors (e.g., culture, personality, resources, individual behavior) and environmental factors (e.g., family, friends, community, religion) can potentially help to further define an individual patient’s treatment risks and benefits. Combining biological, clinical, and personal data with environmental information is critical for targeted decisions Algorithms should be developed to classify individuals into subpopulations according to the severity of the disease and the response to specific treatments, by susceptibility to bleeding and/or thrombosis (including recurrence), and/or strategies to mitigate these risks [46].

This nationwide survey provides an updated overview of physicians’ attitudes toward VTE prophylaxis and PE management, identifying both consensus practices and unresolved controversies that warrant targeted investigation. This study has limitations. Most participants were from Italy, and many had attended prior ad hoc educational sessions, which may have influenced their responses as well as contributed to the high degree of consensus observed. Finally, the small number of participants in our survey may limit the generalizability of the findings to broader clinical practice.

## Figures and Tables

**Table 1:** Details of survey participants, including medical specialty, years of experience, and country of practice.

Info	Maximal Agreement (%) per Question (Q) and Details of Survey Respondents.										
Medical Specialty	Q1.1 (93.44)	Q1.2 (91.26)	Q2 (80.46)	Q3 (77.01)	Q4 (82.78)	Q5 (88.57)	Q6 (77.97)	Q7 (80.09)	Q8 (82.46)	Q9 (93.99)	Q10 (70.93)
Int. Med.	135	129	119	111	127	124	91	99	107	130	103
Neurol.	10	10	6	7	2	7	7	11	9	11	3
Ob/Gyn.	2	2	0	0	2	2	2	2	0	2	2
Ger.	8	8	5	3	4	7	1	7	8	8	4
Cardiol.	6	9	4	5	3	7	7	7	8	8	5
GE.	1	1	1	1	1	2	1	1	1	2	0
Anesth.	1	1	0	1	1	1	2	1	1	1	1
Orthop.	0	0	0	0	0	1	3	1	0	1	0
GP.	1	0	0	0	1	0	2	0	1	1	0
Other	7	7	5	6	8	4	22	15	6	8	4
Total	171	167	140	134	149	155	138	144	141	172	122
**Country of** **residence**											
Italy	149	145	125	124	130	141	103	128	123	147	108
No-Italy	22	22	15	10	19	14	35	16	18	25	14
**Years of** **experience**											
>15 yrs	109	109	83	80	90	99	82	86	90	110	75
<15 yrs	57	53	54	51	54	50	48	54	47	57	44
Missing	5	5	3	3	5	6	8	4	4	5	3

Abbreviation: Q, question; Int. Med: Internal Medicine; Neurol: Neurology; Ob/Gyn: Obstetrics and Gynecology; GE: Gastroenterology; Ger: Geriatrics; Cardiol: Cardiology; Anesth: Anesthesiology; Orthop: Orthopedic Surgery; GP: general practitioner.

**Table 2:** Participant agreement (%) reached in the multiple-choice questionnaire.

Question	Choices	Appropriate N, (%)	Uncertain N, (%)	Inappropriate N, (%)	Total N
1.1 VTE risk assessment should always be performed in hospitalized medical patients	None	171 (93.44)	9 (4.92)	3 (1.64)	183
1.2 Bleeding risk assessment should always be performed in hospitalized medical patients	None	167 (91.26)	12 (6.56)	4 (2.19)	183
**Consensus:** Participants (>90%) agreed that VTE risk assessment and bleeding-risk assessment should be performed in hospitalized medical patients.					
2. Which VTE prophylaxis would you prescribe in an elderly patient with stage IV chronic renal failure, (clearance 22 mL/min), a Padua score > 4 and an IMPROVE bleeding score < 7 hospitalized for an acute ischemic stroke?	Fondaparinux 1.5 mg	92 (52.27)	29 (16.48)	55 (31.25)	176
	Enoxaparin 2000 IU	114 (65.14)	36 (20.57)	25 (14.29)	175
	Enoxaparin 4000 IU	18 (10.34)	16 (9.2)	140 (80.46)	174
	aPTT-adjusted calcium heparin	96 (53.33)	30 (16.67)	54 (30)	180
Consensus: In patients with severe CRF (creatinine clearance < 30 mL/min), participants agreed that low-dose enoxaparin (2000 IU) was better than aPTT-adjusted doses of calcium heparin for prevention of DVT.					
3. A patient taking aspirin plus clopidogrel (dual antiplatelet therapy [DAPT]) for an NSTEMI that occurred >12 months earlier is hospitalized for heart failure. He has a Padua score > 4 and an IMPROVE bleeding score < 7. Which medical prophylaxis for VTE would you prescribe to him?	Maintain DAPT and initiate sc enoxaparin therapy at a standard dose of 4000 IU sc od	62 (35.23)	24 (13.64)	90 (51.14)	176
	Maintain DAPT and initiate sc enoxaparin at a reduced dose of 2000 IU od	10 (5.75)	30 (17.24)	134 (77.01)	174
	Stop aspirin, maintain clopidogrel and add enoxaparin at a standard dose of 4000 IU SC od	65 (37.14)	40 (22.86)	70 (40)	175
	Stop clopidogrel, maintain aspirin, add enoxaparin 4000 IU od	115 (64.25)	26 (14.53)	38 (21.23)	179
Consensus: Participants agreed that maintaining DAPT and initiating therapy with enoxaparin at a reduced dose of 2000 IU sc od was inappropriate for acute DVT prevention in patients on DAPT hospitalized for heart failure.					
4. A patient with active lung cancer is hospitalized because of pneumonia. He has a Padua score > 4 and an IMPROVE bleeding score < 7. Which management approach would you consider?	The Khorana should be used to decide the strategy	106 (60.57)	32 (18.29)	37 (21.14)	175
	Administer fondaparinux at prophylactic dose	110 (62.5)	32 (18.18)	34 (19.32)	176
	Administer enoxaparin at prophylactic dose	149 (82.78)	24 (13.33)	7 (3.89)	180
	Administer apixaban 2.5 mg bid	45 (25.42)	24 (13.56)	108 (61.02)	177
	Decide the strategy without considering the Khorana score	37 (21.14)	34 (19.43)	104 (59.43)	175
Consensus: Participants agreed to administer enoxaparin (82.78%) as the treatment of choice for acute DVT prevention in a patient with active lung cancer hospitalized because of pneumonia.					
5. Thromboprophylaxis in a 21 weeks’ pregnant 35-year-old woman on low-dose aspirin for gestational hypertension, hospitalized for ≥3 days for urgent clinical evaluations. How would you manage her risk?	Thromboprophylaxis (high risk of VTE)	95 (54.91)	24 (13.87)	54 (31.21)	173
	Consider thromboprophylaxis (intermediate risk of VTE)	73 (41.48)	45 (25.57)	58 (32.95)	176
	Thromboprophylaxis to be initiated if additional VTE risk factors are present (low thromboembolic risk)	61 (34.66)	26 (14.77)	89 (50.57)	176
	Administer apixaban (2.5 mg bid)	10 (5.71)	10 (5.71)	155 (88.57)	175
	She should avoid dehydration and prolonged bed rest	55 (31.07)	19 (10.73)	103 (58.19)	177
6. Which acute therapy would you prescribe for a patient with intermediate-high risk of PE, according to the ESC, who does not quite meet the hemodynamic instability criteria (e.g., SBP 100 mmHg)?	iv thrombolysis	17 (9.77)	28 (16.09)	129 (74.14)	174
	iv UFH	89 (50)	40 (22.47)	49 (27.53)	178
	sc fondaparinux/LMWH at therapeutic dose	138 (77.97)	20 (11.3)	19 (10.73)	177
	Oral rivaroxaban 15 mg bid	90 (51.14)	31 (17.61)	55 (31.25)	176
Consensus: Participants agreed that sc therapeutic doses of fondaparinux/LMWH (77.97%) was the treatment of choice for a patient with intermediate–high-risk PE according to the ESC who does not quite meet the hemodynamic instability criteria.					
7. Which acute therapy would you prescribe for a patient with PE classified at intermediate-low risk according to the ESC?	iv UFH	37 (21.39)	33 (19.08)	103 (59.54)	173
	sc fondaparinux/LMWH at therapeutic dose	144 (80.9)	19 (10.67)	15 (8.43)	178
	Oral rivaroxaban 15 mg bid	126 (71.59)	23 (13.07)	27 (15.34)	176
	Oral edoxaban 60 mg od	54 (31.03)	27 (15.52)	93 (53.45)	174
Consensus: Participants agreed that administering sc therapeutic doses of fondaparinux/LMWH (80.9%) was the treatment of choice for a patient with intermediate–low risk PE, according to the ESC.					
8. Which acute therapy would you prescribe for a patient with mild symptoms and a finding of subsegmental pulmonary embolism associated with moderate hypoxemia (PaO_2_ 75 mmHg)?	Fondaparinux/LMWH at therapeutic dose	138 (78.86)	15 (8.57)	22 (12.57)	175
	Fondaparinux/LMWH at prophylactic dose	10 (5.85)	20 (11.7)	141 (82.46)	171
	Oral rivaroxaban 15 mg bid	112 (64)	29 (16.57)	34 (19.43)	175
	O_2_ therapy only (no anticoagulant treatment)	13 (7.56)	19 (11.05)	140 (81.4)	172
Consensus: Participants agreed that sc prophylactic doses of fondaparinux or LMWH (78.86%) were inappropriate for a patient with mild symptoms of subsegmental PE associated with moderate hypoxemia (PaO_2_ 75 mmHg).					
9. Which is the optimal approach to manage a patient who has been correctly treated for PE; and has normal arterial blood gas (ABG) analysis with persistent dyspnea 3 months later?	Evaluate echocardiogram	172 (93.99)	9 (4.92)	2 (1.09)	183
	Run VQ scan	110 (60.44)	53 (29.12)	19 (10.44)	182
	Conduct cardiopulmonary exercise tests	95 (51.91)	60 (32.79)	28 (15.3)	183
	Reassure the patient that dyspnea will disappear over time	20 (10.99)	49 (26.92)	113 (62.09)	182
Consensus: Participants agreed that echocardiogram (93.99%) was the optimal approach to manage a patient who has been correctly treated for PE and has normal ABG analysis with persistent dyspnea 3 months later.					
10. When deciding whether to discharge a patient with PE, which of the following applies?	0 sPESI suffices	36 (20.81)	45 (26.01)	92 (53.18)	173
	Decision based on the Hestia criteria	52 (30.77)	58 (34.32)	59 (34.91)	169
	Right ventricle dysfunction requires hospitalization regardless of the Hestia criteria	122 (70.93)	29 (16.86)	21 (12.21)	172
Consensus: Only 70.93% of participants (i.e., no consensus) agreed that RVD requires hospitalization regardless of the Hestia criteria when deciding whether to discharge a patient with pulmonary embolism.					

Abbreviations: aPTT, activated partial thromboplastin time; bid, twice a day; DAPT, dual antiplatelet therapy; ESC, European Society of Cardiology; NSTEMI, non-ST elevation myocardial infarction; O_2_, oxygen; od, once a day; PaO_2_, partial pressure of oxygen; PE, pulmonary embolism; SBP, systolic blood pressure; sPESI, simplified Pulmonary Embolism Severity Index; UFH, unfractionated heparin; VQ, ventilation/perfusion; VTE, venous thromboembolism.

## Data Availability

The original contributions presented in this study are included in the article/Supplementary Materials. Further inquiries can be directed to the corresponding authors.

## Supplementary Materials

The following supporting information can be downloaded at https://www.mdpi.com/article/10.3390/jcm14238531/s1, Table S1. 10-statements multiple-choice questionnaire; Table S2. PADUA and IMPROVE scores to assess benefit vs risk in medically ill patients; Table S3. Defining the dose of a drug to be administered based on its renal excretion rate: pathophysiological background; Table S4. Perioperative thromboprophylaxis in subjects chronically treated with antiplatelet agents alone or in combination: 2018 European Recommendations; Table S5. Khorana score to predict risk of VTE for cancer patients based on type of cancer and other factors; Table S6. VTE prophylaxis options in cancer patients: ESMO 2023 recommendations, Table S7. RCOG recommendations for obstetric thromboprophylaxis: risk assessment and management in individuals with a history of VTE and/or thrombophilia, Table S8. Patients with isolated subsegmental PE (SSPE) who should not receive anticoagulation; Table S9. Identifying PE cases eligible for outpatient treatment; Table S10. Independent predictors of VTE recurrence.
