# Supplementary material for: Preferences Among Expert Physicians in Areas of Uncertainty in Venous Thromboembolism Management: Results from a Multiple-Choice Questionnaire
Source: J Clin Med. 2025 Dec 1;14(23):8531. doi: 10.3390/jcm14238531 (PMC12692854; doi:10.3390/jcm14238531)
Supplement: Supplementary file 1 [file jcm-14-08531-s001.zip › jcm-3963461-supplementary.pdf]

Supplementary Materials

Table S1. 10-statements multiple-choice questionnaire.

Question 1.1: **VTE risk assessment should be always performed on hospitalized medical patients.**				
**Choice**	**Appropriate (%)**	**Uncertain (%)**	**Inappropriate (%)**	**Total participants**

Question 1.2: **Bleeding risk assessment should be always performed on hospitalized medical patients.**				
**Choice**	**Appropriate (%)**	**Uncertain (%)**	**Inappropriate (%)**	**Total participants**

Suggested reading: *Lancet 2008* Feb 2;371(9610):387-94; *J.Thromb Haemost.* 2022 Feb;20(2):409-21; *J. Thromb Thrombolysis.* 2016 Oct;42(3):336-9; *Blood.* 2020 May 14;135(20):1788-810; *Blood Adv.* 2023 May 9;7(9):1671; *BMJ Open.* 2021 Jul 29;11(7): e045672.

Question 2: **Which VTE prophylaxis would you prescribe in an elderly patient with stage IV chronic renal failure (CRF, clearance 22 mL/min), a Padua score >4 and an IMPROVE bleeding score <7 who has been hospitalized for an acute ischemic stroke?**				
**Choice**	**Appropriate (%)**	**Uncertain (%)**	**Inappropriate (%)**	**Total participants**
Enoxaparin 2000 IU				
Enoxaparin 4000 IU				
aPTT-adjusted calcium heparin				

Suggested reading: *Thromb Haemost. 2009 May;101(5):893-901; Lancet. 2020 Nov 28;396(10264):1767-76; J Clin Pharmacol. 2007 Feb;47(2):218-26; Chest. 2012 Feb;141(2 Suppl): e419S-e496S; Am J Nephrol. 2024;55(2):146-64; Blood Adv. 2018 Nov 27;2(22):3198-225.*

Question 3: **A patient taking aspirin plus clopidogrel (dual antiplatelet therapy, DAPT) for a NSTEMI that occurred >12 months earlier is hospitalized for heart failure. He has a Padua score >4 and an IMPROVE bleeding score <7. Which medical prophylaxis for VTE would you prescribe to him?**				
**Choice**	**Appropriate (%)**	**Uncertain (%)**	**Inappropriate (%)**	**Total participants**
Maintain DAPT and initiate enoxaparin therapy at a standard dose of 4000 IU sc od				
Maintain DAPT and initiate therapy with enoxaparin at a reduced dose of 2000 IU sc od				
Stop aspirin, maintain clopidogrel and add enoxaparin at a standard dose of 4000 IU sc od				

Stop clopidogrel, maintain aspirin, add enoxaparin 4000 IU sc od				

Suggested reading: *Circulation.* 2012 Sep 4;126(10):1185-93; *Blood Rev.* 2015 Sep;29(5):335-43; *Eur Heart J Qual Care Clin Outcomes.* 2016 Jul 1;2(3):172-83; *N Engl J Med.* 2015 May 7;372(19):1791-800; *N Engl J Med.* 2014 Dec 4;371(23):2155-66; *Eur J Anaesthesiol.* 2018 Feb;35(2):77-83; *J Thromb Haemost.* 2022 Oct;20(10):2226-36.

Question 4: **A patient with active lung cancer is hospitalized because of pneumonia. He has a Padua score >4 and an IMPROVE bleeding score <7. Which management approach would you consider?**

**Choice**	**Appropriate (%)**	**Uncertain (%)**	**Inappropriate (%)**	**Total participants**
The Khorana should be used to decide the strategy				
Administer fondaparinux at prophylactic dose				
Administer enoxaparin at prophylactic dose				
Administer apixaban 2.5 mg bid				
Decide the strategy without considering the Khorana score				

Suggested reading: *J Clin Oncol.* 2014 Jun 10;32(17):1792-6; *J Thromb Haemost.* 2010 Nov;8(11):2450-7; *N Engl J. Med*. 2013 May 16;368(20):1945-6; *Am J Med.* 2014 Jul;127(7): e33; *Am J Hematol.* 2017 Jun;92(6):501-7; *Ann Oncol*. 2023 May;34(5):452-67.

Question 5: **Thromboprophylaxis in a 21 weeks' pregnant 35-year-old woman on low-dose aspirin for gestational hypertension, hospitalized for ≥3 days for urgent clinical evaluations. How would you manage her risk?**

**Choice**	**Appropriate (%)**	**Uncertain (%)**	**Inappropriate (%)**	**Total participants**
Thromboprophylaxis (high risk of VTE)				
Consider thromboprophylaxis (intermediate risk of VTE)				
Thromboprophylaxis to be initiated if additional VTE risk factors there are present (low thromboembolic risk)				
Administer apixaban (2.5 mg bid)				
She should avoid dehydration and prolonged bed rest				

Suggested reading: *Semin Perinatol.* 2019 Jun;43(4):222-8; *Obstet Gynecol.* 2020 Jun;135(6):1492-5; *Front Cardiovasc Med.* 2022; 9:1073148.

Question 6: **Which acute therapy would you prescribe for a patient with intermediate-high risk of PE, according to the ESC, who does not quite meet the hemodynamic instability criteria (e.g., SBP 100 mmHg)?**

**Choice**	**Appropriate (%)**	**Uncertain (%)**	**Inappropriate (%)**	**Total participants**
Intravenous thrombolysis				
Intravenous unfractionated heparin				
sc fondaparinux/LMWH (therapeutic doses)				
Oral rivaroxaban 15 mg bid				

Suggested reading: *N Engl J Med.* 2014 Apr 10;370(15):1402-11; *Eur Heart J.* 2018 Dec 14;39(47):4186-95; *Thorax. 2018* May;73(5):464-71; *Eur Heart J.* 2020 Jan 21;41(4):543-603; *J Thromb Haemost.* 2022 Feb;20(2):409-21; *Thromb Haemost.* 2023 Jun;123(6):613-26.

Question 7: **Which acute therapy would you prescribe for a patient with PE classified at intermediate-low risk according to the ESC?**				
**Choice**	**Appropriate (%)**	**Uncertain (%)**	**Inappropriate (%)**	**Total participants**
iv unfractionated heparin iv				
sc fondaparinux/LMWH (therapeutic dose)				
Oral rivaroxaban 15 mg bid				
Oral edoxaban 60 mg od				

Suggested reading: *J Thromb Haemost.* 2022 Feb;20(2):409-21.

Question 8: **Which acute therapy would you prescribe for a patient with mild symptoms and a finding of subsegmental PE (SSPE) associated with moderate hypoxemia (PaO_2 75 mmHg)?**				
**Choice**	**Appropriate (%)**	**Uncertain (%)**	**Inappropriate (%)**	**Total participants**
Fondaparinux/LMWH at therapeutic dose				
Fondaparinux/LMWH at prophylactic dose				
Oral rivaroxaban 15 mg twice daily				
O_2 therapy only (no anticoagulation)				

Suggested reading: *Clin Appl Thromb Hemost.* 2012 Jan-Feb;18(1):20-6; *Ann Intern Med.* 2022 Jan;175(1):29-35.

Question 9: **Which is the optimal approach to manage a patient who has been appropriately treated for PE; and has normal arterial blood gas (ABG) analysis with persistent dyspnea 3 months later?**				
**Choice**	**Appropriate (%)**	**Uncertain (%)**	**Inappropriate (%)**	**Total participants**
Evaluate echocardiogram				
Conduct VQ scan				
Conduct cardiopulmonary exercise tests				
Reassure the patient that dyspnea will disappear over time				

Suggested reading: *Blood Rev.* 2014 Nov;28(6):221-6; *J Thromb Haemost.* 2014 Apr;12(4):459-68; *Circulation*. 2014 Jan 28;129(4):479-86; *Chest*. 2017 May;151(5):1058-68; *Thromb Res.* 2018 Apr; 164:157-62; *Eur Heart J.* 2022 Sep 21;43(36):3387-98.

Statement 10: **When deciding whether to discharge a patient with PE, which of the following applies?**				
**Choice**	**Appropriate (%)**	**Uncertain (%)**	**Inappropriate (%)**	**Total participants**
0 sPESI suffices				
Decision based on the Hestia criteria				

Right ventricle dysfunction (RVD) requires hospitalization regardless of the Hestia criteria				

Suggested reading: *J Thromb Haemost.* 2013 Apr;11(4):686-92; *Thromb Res.* 2014 May;133 Suppl 2:S10-6; *Eur Heart J.* 2019 Mar 14;40(11):902-10; *Eur Heart J.* 2020 Jan 21;41(4):543-603; *Eur Heart J.* 2021 Aug 31;42(33):3190-9; *Eur Respir J.* 2021 Feb;57(2).

Abbreviations: ABG, arterial blood gas; aPTT, activated partial thromboplastin time; bid, twice a day; CRF, chronic renal failure; DAPT, dual antiplatelet therapy; DVT, deep vein thrombosis; ESC, European Society of Cardiology; iv, intravenous; LMWH, low molecular weight heparin; NSTEMI, non-ST-elevation myocardial infarction; od, once a day; PE, pulmonary embolism; PCO_2, partial pressure of carbon dioxide; PO_2, partial pressure of oxygen; SBP, systolic blood pressure; sc, subcutaneous; VQ, ventilation/perfusion; VTE, venous thromboembolism.

Table S2. PADUA and IMPROVE scores to assess benefit vs risk in medically ill patients. ^

A) Assessing VTE RISK: PADUA Risk Score[1]

VTE risk factors[1]	Score
Active cancer	3
Previous VTE	3
Reduced mobility	3
Already known thrombophilia condition(s)	3
Recent (< 1 month) trauma and/or surgery	2
Elderly age (> 70 years)	1
Heart and/or respiratory failure	1
Acute myocardial infarction or ischemic stroke	1
Acute infection and/or rheumatological disorder	1
Obesity (BMI > 30 kg/m^2)	1
Ongoing hormonal treatment	1
Scores ≥ 4 indicate high risk of VTE.	

B) Assessing BLEEDING RISK: IMPROVE Bleeding Score[1,2]

Bleeding risk factors [1]	Score
Renal failure (GFR 30–59 vs ≥ 60 mL/min/1.73 m^2)	1
Male vs female	1
Age 40–80 vs < 40 years	1.5
Current cancer	2
Rheumatic disease	2
Central venous catheter	2
ICU/CCU stay	2.5
Renal failure (GFR < 30 vs ≥ 60 mL/min/1.73 m^2)	2.5
Hepatic failure (INR > 1.5)	2.5
Age ≥ 85 vs < 40 years	3.5
Platelet count < 50 × 10^9/L	4
Bleeding in 3 months before admission	4
Active gastroduodenal ulcer	4.5
Scores ≥7 indicate high risk of bleeding.	

^ Modified from: [1]: Schunemann et al. *Blood Adv.* 2018;2(22):3198–3225, [2]: Decousus et al. *Chest.* 2011;139(1):69–79.

Abbreviations: BMI, body mass index; CCU, critical care unit; GFR, glomerular filtration rate; ICU, intensive care unit; INR, international normalized ratio, VTE, venous thromboembolism.

Table S3. Defining the dose of a drug to be administered based on its renal excretion rate: pathophysiological background. [§]

Pathophysiological Background	Examples	Actions required
**High renal excretion**	**ACE inhibitors**<br>Perindopril and lisinopril do require dosage adjustment (renal excretion).	Fosinopril and ramipril do not require large dose modifications (hepatobiliary excretion).
	**HUMAN INSULIN, ORAL HYPOGLYCEMIC AGENTS**	Metformin does require large dose modifications<br><br>Glipizide, pioglitazone, linagliptin do not require large dose modifications (hepatic metabolism).
	**HEPARINS**<br>*Some LMWH* (enoxaparin, bemiparin, certoparin) and *fondaparinux* are excreted renally and require dosage adjustment in patients with a CrCl <30 ml/min. [a]	**Unfractionated heparin** (calcium or sodium) is metabolized in the liver and at endothelial level and does not require dose modifications.
**Distribution and Excretion changes**<br>↓<br>**Effects on drug concentration**	**DOACs**<br>-APIXABAN excretion (75% in the feces, 25% by the kidney)<br>-RIVAROXABAN excretion (33% in the feces, 66% by the kidney).	Apixaban: does not require dose modifications.<br>Rivaroxaban: does require large dose modifications.
	**DIGOXIN** excreted by renal filtration and renal secretion; the volume of distribution can be reduced because of 50% reduction of the tissue binding sites of digoxin embodied by Na and K ATPase pumps.[b]	Dose reduction to avoid the risk of toxicity (narrow therapeutic index)
	**BETA-BLOCKERS**<br>-Atenolol, Sotalol:[b] urinary excretion; → reduce doses in CRF<br>-Bisoprolol: urinary excretion; →dose reduction in advanced CRF	Metoprolol and propranolol do not require dose modifications (hepatic metabolism).
	**AMIODARONE:** metabolized in the liver, protein binding: 99%	Amiodarone does not require dose modification.
**Increase in gastric pH.**<br>↓<br>**absorption changes**	**FUROSEMIDE, IRON**<br>↓<br>Low absorption, low bioavailability (≈20% and 50% respectively)	Dose increase often needed.
**Low renal excretion**	**LITHIUM** urinary excretion by filtration followed by tubular reabsorption[c]	Dose reduction needed (to avoid accumulation/neurotoxic effects).[d]

[§]Modified from: *Clin J Am Soc Nephrol* 2018; 13: 1085-1095; *Clin J Am Soc Nephrol* 2019; 14 (5): 757-764.

Abbreviations: ACE, angiotensin-converting enzyme; CrCl, creatinine clearance; CRF: chronic renal failure; K, potassium; LMWH, low molecular weight heparin; Na, sodium.

[a]In a systemic review of 674 publications, no accumulation was observed in studies of dalteparin or tinzaparin; [b]Sotalol has renal excretion, not bound to proteins, mandatory to reduce its dose in CRF; [c]Furosemide increases lithium excretion by reducing its tubular absorption; [d]Secretion inhibitors (e.g., amiodarone or carvedilol) can act at this level.

Table S4. Perioperative thromboprophylaxis in subjects chronically treated with antiplatelet agents alone or in combination: 2018 European Recommendations. §

In patients receiving antiplatelet agents chronically, we recommend thromboprophylaxis in cases of moderate/high VTE risk, whilst assessing the risk of perioperative bleeding (grade 1B).
In patients receiving antiplatelet agents chronically, if the risk of VTE outweighs the risk of bleeding, we suggest pharmacological (anticoagulant) prophylaxis (LMWH, direct oral anticoagulants, fondaparinux depending on the indication) (grade 2C).
In patients treated with DAPT undergoing a procedure associated with a high risk of VTE, we suggest resuming antiplatelet agents shortly after the procedure, prioritizing over pharmacological VTE prevention (grade 2C).
If an anticoagulant is associated with an antiplatelet agent, we suggest the administration of the lowest approved dose (grade 2C).
If the risk of bleeding of a combination of an antiplatelet agents and an anticoagulant outweighs the risk of VTE, we suggest considering intermittent pneumatic compression over anticoagulant prophylaxis, without discontinuing the antiplatelet agents (grade 2C).
Patients in whom neuraxial anesthesia is planned, although the administration of aspirin alone does not increase the incidence of spinal hematoma, a higher rate of complications could appear if pharmacological thromboprophylaxis is administered concurrently. In these cases, postoperative thromboprophylaxis initiation should be suggested (grade 2C).
After surgery, the first dose of aspirin should be given as soon as possible, once hemostasis is considered adequate (in general, the day after surgery; grade 2B). In the case of clopidogrel, the main recommendation is to give the drug without any loading dose between 24 and 48 h after surgery (grade 2C).
Monitoring for clinical signs of bleeding or unexplained anemia is recommended during concomitant administration of an anticoagulant for thromboprophylaxis (LMWH, unfractionated heparin, fondaparinux, warfarin or any other) and an antiplatelet agent throughout the postoperative period (grade 1C).
Nonsteroidal anti-inflammatory drugs should be avoided in patients treated with antiplatelet agents (grade 2C).

§Modified from: *Eur. Journal of Anaesthesiology* 2018;35(2):139-141.

Abbreviations: DAPT, dual antiplatelet therapy; LMWH, low molecular weight heparin; VTE, venous thromboembolism.

Table S5. Khorana score to predict risk of VTE for cancer patients based on type of cancer and other factors[§]

**Variable**		**Risk score**
Very high-risk tumor (stomach, pancreas)		2
High-risk tumor (lung, gynecological, genitourinary excluding prostate)		1
Hemoglobin level < 100 g/L or use of red cell growth factors		1
Pre-chemotherapy leucocyte counts 11 x 10^9/l		1
Pre-chemotherapy platelet count ≥350 x 10^9/l		1
BMI ≥35 kg/m^2		1
Total score	Risk category	Risk of symptoms
0	Low	0.3-0.5%
1-2	Intermediate	1.8-4.8%
≥3	high	6.7-12.9%

[§] Modified from: *Ann Oncol.* 2023 May;34(5):452-467.
Abbreviations: BMI, body mass index; VTE, venous thromboembolism.

Table S6. VTE prophylaxis options in cancer patients: ESMO 2023 recommendations§.

Options	Hospitalized patients	Surgical patients	Ambulatory patients
**Heparins[a]**			
UFH	5000 IU every 8 h	5000 IU 2-4 h preop and then every 8 h	-
Bemiparin	3500 anti-Xa IU od	3500 IU anti-Xa starting 2 h preoperatively or 6 h postoperatively and 3500 IU anti-Xa od thereafter	3500 IU anti-Xa od[b]
Dalteparin	5000 anti-Xa IU od	5000 IU anti-Xa 12 h preoperatively and 5000 IU anti-Xa od thereafter	5000 IU anti-Xa od[b,c]
Enoxaparin	4000 anti-Xa IU od	4000 IU anti-Xa 12 h preoperatively and 4000 IU anti-Xa od thereafter	4000 IU anti-Xa od[b]
Nadroparin	3800 anti-Xa IU od.<br>(if weight >70 kg, 5700 anti-Xa IU/kg od)	2850 IU anti-Xa 2–4 h preoperatively and 2850 IU anti-Xa od thereafter	3800 IU anti-Xa od (if weight >70 kg, 5700 anti-Xa IU od) [b]
Tinzaparin	4500 anti-Xa IU od	4500 IU anti-Xa od, beginning 12 hours post-operatively	4500 IU anti-Xa od[b]
**Selective parenteral factor Xa inhibitor**			
Fondaparinux	2.5 mg od	2.5 mg od beginning 6–8 hours post-operatively	No studies in the outpatient setting
**DOACs**			
Apixaban	*Not recommended*	*Not recommended*	2.5 mg orally bid[b]
Rivaroxaban	*Not recommended*	*Not recommended*	10 mg orally od[b]
**Mechanical prophylaxis**			
IPC	If pharmacological prophylaxis is contraindicated[d]	*If pharmacological prophylaxis is contraindicated[d]*	*Not recommended*
Venous foot pump			*Not recommended*
GCS			*Not recommended*

§Modified from: *Ann Oncol.* 2023 May;34(5):452-467.

Abbreviations: bid, twice daily; DOAC, direct oral anticoagulant; GCS, graduated compression stockings; IPC, intermittent pneumatic compression; od, once daily; UFH, unfractionated heparin; VTE, venous thromboembolism.

[a]Approved indications and dosages of anticoagulants may vary across different countries; [b]Lack of a specific indication for cancer outpatients in the package inserts; DOACs are not registered for use in ambulatory patients in Italy; [c]In pancreatic cancer, higher doses have been used in clinical trials in this setting; [d]Some patients may also have a contraindication for mechanical prophylaxis (e.g., patients with peripheral limb ischemia).

Table S7. RCOG recommendations for obstetric thromboprophylaxis: risk assessment and management in individuals with a history of VTE and/or thrombophilia[§]

**Risk factors**	**Antenatal (AN)**	**Postnatal (PN)**
<u>High Risk:</u><br>Prophylaxis from first trimester and 6 weeks PN	Any previous VTE (except single event with previous surgery)	Any previous VTE<br>Antenatal LMWH<br>High risk thrombophilia with/without previous VTE<br>Low risk thrombophilia and family history
<u>Intermediate risk:</u><br>Consider AN prophylaxis and at least 10 days PN prophylaxis	Hospital admission<br>High risk of thrombophilia with no VTE<br>Single previous VTE with surgery<br>Medical comorbidity<br>Surgical procedure<br>Ovarian hyperstimulation syndrome	Caesarean section in labor<br>BMI >40 kg/m^2<br>Readmission or prolonged admission in puerperium<br>Surgical procedure except immediate perineal repair<br>Medical comorbidity
<u>Co-existence of low-risk factors</u>:<br>*4 or more*:<br>prophylaxis from first trimester.<br>*3 or more AN; 2 or more PN*:<br>prophylaxis from 28 weeks.<br>*≤3 AN; ≤2and PN*:<br>mobilization, avoid dehydration.	BMI > 30kg/m^2<br>Age >35 years<br>Parity 3 or more (not including current)<br>Smoker<br>Gross varicose veins<br>Immobility e.g., paraplegia, pelvic pain<br>Family history of unprovoked or estrogen provoked VTE in 1st degree relative<br>Low risk thrombophilia<br>Multiple pregnancy<br>IVF/ART	Same as Antenatal with:<br>Elective caesarean section<br>Current systemic infection<br>Immobility: paraplegia, pelvic girdle pain, long distance travel<br>Current pre-eclampsia<br>Delivery less than 37 weeks<br>Stillbirth in this pregnancy<br>Mid-cavity rotational or operative delivery<br>Prolonged labor >24 h<br>PPH >1 liter or blood transfusion

Examples

**Risk**	**Type of thrombophilia**	**Recommended management**
Very high	Previous VTE on anti-coagulant therapy,<br>Antithrombin deficiency.	AN: high dose LMWH (for example, enoxaparin 40 mg bid or 80 mg od), and PN 6 weeks LMWH (unless restarted on previous anticoagulation)

High	Any previous VTE, except single VTE related to major surgery, antiphospholipid syndrome + previous VTE.	Prophylactic LMWH (e.g., enoxaparin 40 mg od) from first trimester and 6 weeks PN
Intermediate	Homozygous factor V Leiden, protein S or C deficiency, no VTE. Single previous VTE related to major surgery (no family history or other risk factors).	AN: Consider prophylactic dose LMWH and 6 weeks PN prophylactic dose LMWH. AN: Consider prophylactic dose LMWH, recommend prophylactic dose LMWH from 28 weeks gestation and 6 weeks prophylactic dose LMWH PN
Low	Asymptomatic low-risk thrombophilia (prothrombin gene mutation/heterozygous factor V Leiden)	10 days prophylactic dose PN LMWH (consider 6 weeks prophylactic dose PN LMWH if significant family history)

§ Modified from: *Semin. Perinatol*. 43, 2019, 222-228.

Abbreviations: AN, antenatal; ART, assisted reproductive techniques; BMI, body mass index; IVF, *in vitro* fertilization; PN, postnatal; PPH, postpartum hemorrhage, LMWH, low-molecular weight heparin; VTE, venous thromboembolism.

Table S8. Patients with isolated subsegmental PE (SSPE) who should not receive anticoagulation. §

• Good pulmonary–respiratory reserve*
• Neither evidence of DVT with serial leg tests nor history of DVT
• No malignancy
• A transient (reversible) major risk factor for PE that is no longer present.
• A compliant and trustworthy patient who would return for serial noninvasive leg tests.

§ Modified from: *Clin Appl Thromb Hemost*. 2012 Jan-Feb;18(1):20-6.
*Pulmonary–respiratory reserve is defined adequate if none of the following are present:

- pulmonary edema,
- right ventricular failure,
- hypotension (systolic pressure <90 mmHg),
- syncope,
- acute tachyarrhythmias,
- respiratory failure shown by severely abnormal spirometry parameters (forced expiratory volume in 1 second <1.0 L or vital capacity <1.5 L), or blood gas measurements (PO_2 <50 mmHg) PCO_2.

Abbreviations: DVT, deep vein thrombosis; PE, pulmonary embolism; PCO_2, partial pressure of carbon dioxide; PO_2, partial pressure of oxygen.

Table S9. Identifying PE cases eligible for outpatient treatment:
Hestia rules to identify patients who can be discharged[§]

Hemodynamic instability?
Need for thrombolysis/embolectomy?
High risk for bleeding?
Oxygen supply to maintain saturation >90%> 24h?
PE diagnosed during anticoagulation treatment?
Intravenous pain medication >24h?
Creatinine clearance ≤ 30 ml/min?
Severe liver impairment?
Pregnancy?
History of heparin-induced thrombocytopenia?

Modified from: *Thromb Res.* 2014 May:133 Suppl 2: S10-6
[§] If at least one of the above questions is answered with yes, the patient cannot be treated at home.
Abbreviations: PE, pulmonary embolism.

Table S10. Independent predictors of VTE recurrence.

**Predictors of VTE**§	**Predictors of VTE recurrence**§
Age,	Increasing patient age
Surgery, trauma/fracture	Increasing BMI
Central vein catheterization or transvenous pacemaker	Active cancer
Active cancer	Neurological disease with leg paresis;
Neurological disease with leg paresis	Lupus anticoagulant or antiphospholipid antibody
Prior superficial vein thrombosis	Antithrombin, protein C, or protein S deficiency
Varicose veins	Persistently increased plasma fibrin D-dimer
Hospital, or nursing home confinement	
WOMEN ONLY	
Oral contraceptive use,	
Pregnancy/ postpartum status	
Hormone therapy	

§Modified from: Schünemann *et al. Blood Adv.* 2018;2(22):3198–3225

Abbreviations: BMI, body mass index; VTE, venous thromboembolism.
